# Experiences of antibiotic use and healthcare access among migrants to the UK: a qualitative study

**DOI:** 10.1186/s12889-025-22384-1

**Published:** 2025-05-15

**Authors:** Luisa Silva, Amani Al-Oraibi, Shajwan Nanakali, Mayuri Gogoi, Osama Hassan, Isra Al-Sharabi, Pankhuri Sahare, Manish Pareek, Irtiza Qureshi, Laura B. Nellums

**Affiliations:** 1 Lifeorgdivision and Population Health, School of Medicine, University of Nottingham, Nottingham, UK; 2 Department of Respiratory Sciences, University of Leicester, Leicester, UK; 3 Development Centre for Population Health, University of Leicester, Leicester, UK; 4 Pharmacy Department, Tishk International University, Erbil, Iraq; 5 Doctors of the World, London, UK; 6 Centre for Public Health and Epidemiology, University of Nottingham, Nottingham, UK; 7 College of Population Health, Health Sciences Center, University of New Mexico, Albuquerque, NM USA; 8 Centre for Inequalities, Institute for Lifecourse Development, University of Greenwich, London, UK

**Keywords:** Migrant health, Global health, Antibiotic knowledge, Antibiotic use, Antimicrobial resistance, Qualitative research, Health inequalities, Healthcare access

## Abstract

**Background:**

In recent years, migration to and within Europe has increased. Human mobility has been hypothesised as a contributing factor towards antimicrobial resistance (AMR). However, there is limited evidence to explain how migration contributes towards antibiotic resistance. More qualitative research regarding migrants’ perspectives of antibiotic use is needed to understand this complex interaction. The aim of this study was to explore experiences of antibiotic use and healthcare access among migrants in the UK, and how this might influence the risk of AMR.

**Methods:**

Adult migrants were purposively recruited through community organisations, collaborators, online platforms and snowball sampling representing different migrant statuses, countries of origin and ethnicities. Semi-structured interviews were conducted online, by phone or face-to-face, in participants’ preferred languages, between March and July 2022 exploring antibiotic use and healthcare access. Data were analysed thematically and the study was informed by a Project Advisory Committee, with members from Doctors of the World and professionals who were previously refugees or asylum seekers.

**Results:**

Twenty-seven migrants (17 males and 10 females), aged 21–60, from 17 different countries were interviewed. Four main themes were generated: 1) Uncharted territory: navigating a new healthcare system (sub-themes (a) access to care during journey, (b) difficult access to healthcare in the UK and (c) comparison between different healthcare systems); 2) Preserving the sense of agency and decision-making around antibiotic use, 3) Self-perpetuating cycle (sub-themes - (a) co-infections; (b) using alarming symptoms or “red-flags” and (c) taking antibiotics due to previous similar symptoms or persisting symptoms), and 4) The fragile state of the patient-doctor relationship.

**Conclusions:**

These findings give useful insight into barriers faced by migrants when trying to access healthcare services both en route and after arriving in the UK, as well as their attitudes and behaviours in relation to antibiotics. Results also shed light on the complexity of factors contributing to health-seeking behaviour and antibiotic use, and how these may vary depending on previous experiences. We discuss implications for future research and practice, and how current policies may need to evolve to better support and reduce possible risk factors for AMR in migrant communities.

**Supplementary Information:**

The online version contains supplementary material available at 10.1186/s12889-025-22384-1.

## Background

Bacterial antimicrobial resistance (AMR) occurs when bacteria evolve new resistance mechanisms to antibiotic medicines, rendering these drugs ineffective to treat bacterial infections [1]. In 2019, according to the Lancet’s global burden of bacterial antimicrobial resistance report, an estimated 1.27 million deaths were attributed to bacterial AMR [2], with AMR emerging as one of the leading global public health threats of the 21st century. Globalisation and the increasing movement of people worldwide have contributed in part to the dissemination of antibiotic resistant organisms [3]. The factors at play in the relationship between human mobility and AMR are complex and multi-faceted, with travel and hospitalisation abroad playing an important role [4]. However, there is insufficient evidence around the impact of migration on AMR worldwide.

Globally, migration is increasing due to globalisation, climate change, social and economic inequities, and conflict, with 281 million international migrants residing in countries outside their country of birth [5]. In the UK, there are an estimated 9.6 million migrants (representing roughly 14.5% of the population) [6], encompassing a diverse group of individuals such as asylum seekers, refugees, and those migrating for family or economic reasons. Many of these individuals are forcibly displaced, have had difficult or lengthy journeys, or experienced deprivation, poor living conditions, or barriers to accessing timely or appropriate healthcare.

The quantitative data on the subject evidences higher rates of antimicrobial resistance in migrants than the host population in Europe [7]. A clinical study analysing over 14,000 urine samples found that the prevalence of antibiotic resistance was significantly higher among migrants (both refugees and family-reunited), than non-migrant patients [8]. A systematic review of 23 observational studies from Europe [7] reported on antibiotic resistance in 2,319 migrants and found that the pooled prevalence of any AMR carriage or AMR infection in migrants was 25·4% (95% CI 19·1–31·8; I²=98%), including methicillin-resistant Staphylococcus aureus (7·8%, 4·8–10·7; I²=92%) and antibiotic resistant Gram-negative bacteria (27·2%, 17·6–36·8; I²=94%). The pooled prevalence of any AMR carriage or infection was higher in refugees and asylum seekers (33·0%, 18·3–47·6; I²=98%) than in other migrant groups (6·6%, 1·8–11·3; I²=92%). The pooled prevalence of antibiotic-resistant organisms was slightly higher in high-migrant community settings (33·1%, 11·1–55·1; I²=96%) than in migrants in hospitals (24·3%, 16·1–32·6; I²=98%).

The qualitative literature suggests that certain factor may contribute to an increased risk of AMR in some migrant communities, particularly among forced migrants and in high-migrant community settings such as camps and detention centres, though it is important to note the evidence does not suggest an increased risk of onward transmission to receiving communities [7]. Unfamiliarity with new healthcare systems and differences in the accessibility of antibiotics between countries may also be important factors influencing antibiotic use in migrant communities, ultimately impacting on patterns of AMR [9]. In the UK, for example, antibiotics are usually only available with a prescription, though in other countries worldwide antibiotics can still be accessed over-the counter without a prescription. Migrants have reported bringing antibiotics from their countries of origin for their own use and to share with family and friends unable to access antibiotics [10].

Different antibiotic stewardship practices in countries of origin, varied knowledge and expectations regarding antibiotics and the risk of AMR, and differences in the accessibility of care between countries may influence how and when migrant communities utilise antibiotics [11]. Examples of barriers faced by migrants include language, feeling dismissed by healthcare professionals, immigration status, entitlement to statutory healthcare, stigma and discrimination, financial barriers, and limited access to information about how the healthcare system works [12, 13].

Healthcare provider-related factors may also influence patterns of antibiotic use. Indeed, overuse of antibiotics is a significant driver of AMR [14, 15] and several studies have focused on the factors contributing to the inappropriate use of antibiotics in primary care, where the majority of antibiotics is prescribed [16, 17]. Others have suggested possible interventions that may tackle overprescribing, [18, 19]. When it comes to migrant groups, for example, time constraints in consultations, challenges in diagnosing patients or providing adequate explanations around treatment due to linguistic or cultural differences, may contribute to inappropriate prescribing. A desire to build better patient-doctor relationships by building trust and meeting patient expectations, concerns around loss to follow up, or perceived risk of infection may also contribute to overprescribing in migrant communities [20, 21].

In the context of increasing AMR and migration worldwide, and clear evidence of barriers to the provision of adequate and timely care to these populations, there is an urgent need for research to understand the experiences of these communities around antibiotic use in receiving countries in order to inform evidence-based interventions and policy changes. This qualitative study is part of a wider research project, EMERGE (Examining Migration and the Epidemiology of Resistance in Groups in Europe) [22, 23], which aims to generate novel evidence on antimicrobial resistance (AMR) in migrants to Europe. In this paper, our specific objectives were to gain migrants’ perspectives around their experiences of antibiotic use in the UK including: (1) Experience of antibiotic use and healthcare access at all stages of migration journey; (2) their views of the UK healthcare system and how it compares to their country of origin; (3) migrants’ perceptions around antibiotic prescribing and the patient-doctor relationship; and (4) possible factors influencing health-seeking behaviour and antibiotic use in this group of patients.

A second EMERGE study exploring healthcare professionals’ views of prescribing antibiotics within migrant communities is also being undertaken and findings will be available at a later date.

## Methods

Adult migrant participants living in the UK were recruited through community organisations (such as Doctors of the World), collaborators, the Project Advisory Committee, online platforms [22, 23] and snowball sampling by researchers and participants. As we advertised the study through different means, we were not able to collect data on the number of people who may have heard about the study but declined to participate. All participants that provided informed consent completed the study. We adopted a purposeful sampling technique, to recruit from different geographical origins, migration status, ages and genders. Participants were adults (> 18 years), born outside the United Kingdom (UK) and with differing educational qualifications, immigration and work statuses. This diverse sample enabled us to collect a rich nuanced dataset and recruitment was guided by data saturation [24].

Semi-structured in-depth interviews were conducted between March and July 2022 by phone, online (via Microsoft Teams application), or face-to-face in confidential spaces, depending on participants’ preference or logistics. Only the researchers and participants were present during the interviews and one interview only was conducted with each participant. A short questionnaire with demographic questions such as age, ethnicity and country of origin was completed by participants before the interviews. OH, LS, SN, AAO, conducted the interviews. A semi-structured piloted topic guide was developed with input from migrant communities. The topic guide included open-ended questions, and a narrative chronological approach structured from broader to more specific questions. The topic guide included questions around participants’ experience of the journey to the UK, adaptation to the UK healthcare system, knowledge and use of antibiotics; understanding of antibiotic resistance and perceived health needs. Interviews were conducted in the participant’s preferred language (most were conducted in English, but eight conducted in Arabic and one in Kurdish). When conducted in Arabic or Kurdish, two researchers took part in the interview, one directly asking questions and the other translating. All participants were sent a Participant Information sheet with full details of the study aims and methods. Informed consent was provided by all participants prior to data collection and interviews.

Interviews were recorded and transcribed verbatim, either using a professional transcription service or Premiere Pro software 2022. All transcripts were reviewed and double checked for accuracy by the research team, but no transcript was returned to participants for comment or correction. An inductive thematic analysis approach [24] was adopted to analyse the transcripts, and analysis was carried out utilising Microsoft Excel. OH, LS, SK, AAO, conducted the first round of coding. Progress was discussed in weekly meetings and the coding framework was updated as new codes were identified. Thereafter, the coders along with MG, IQ, PS, IAS and LBN identified the final set of themes and sub-themes. Although we did not ask participants for feedback regarding the main themes, a draft summary of the main findings from the study was sent to collaborators from the Project Advisory Committee for comment and feedback.

This study has been reported according to the COREQ guidelines [25]. (see Additional file 1: COREQ checklist).

### Reflexivity

The research study team was comprised of OH (MD MPH), LS (MBChB PhD), SSN (MPH), AAO (MPH), MG (PhD), IQ (PhD), PS (MPH), IW (BSc MSc), MP (MBChB PhD) and LBN (MSc PhD) who were from diverse genders, ethnicities, educational and professional backgrounds with varying migration statuses across the team. Colleagues’ professional backgrounds included medicine, pharmacy, education, public health, anthropology, social work and health policy with previous research experience and training. As a team from diverse personal and professional backgrounds, we reflected on how our own experiences may have affected both data generation and interpretation, through a process of active reflexivity. Field notes were also made after the interviews to complement team discussions.

This was particularly important to consider for some of the interviews - eight of these were conducted in Arabic and one in Kurdish, as two of the researchers were native speakers of these languages. This may have helped positively engage with participants and create a more natural setting for data gathering, but we also acknowledged the importance of trustworthiness and consistency in this process. In other cases, for example for those researchers medically trained, it was important to maintain a focus on data collection rather than a more clinically-nuanced encounter to ensure consistency between interviews.

The team weekly debriefing sessions gave us an insight into the data content from the initial interviews. It allowed us to come across aspects which needed more representation, such as migrants’ living conditions and the influence it held over their health and decisions. Lastly, it helped us become more aware of our personal assumptions and views regarding the research, which ultimately led to strengthening our interview skills and more objectivity in writing the results and findings.

This work was also guided by inputs from the Project Advisory Committee, which includes members from Doctors of the World and professionals who were previously refugees or asylum seekers.

## Results

### Participant characteristics/demographics

A total of 27 participants were recruited and interviewed from diverse regions, ages, ethnicities, migration statuses, educational backgrounds, and time in the UK. Interviews lasted between 21 and 45 min, with a mean duration of 30 min.

**Fig. 1:**
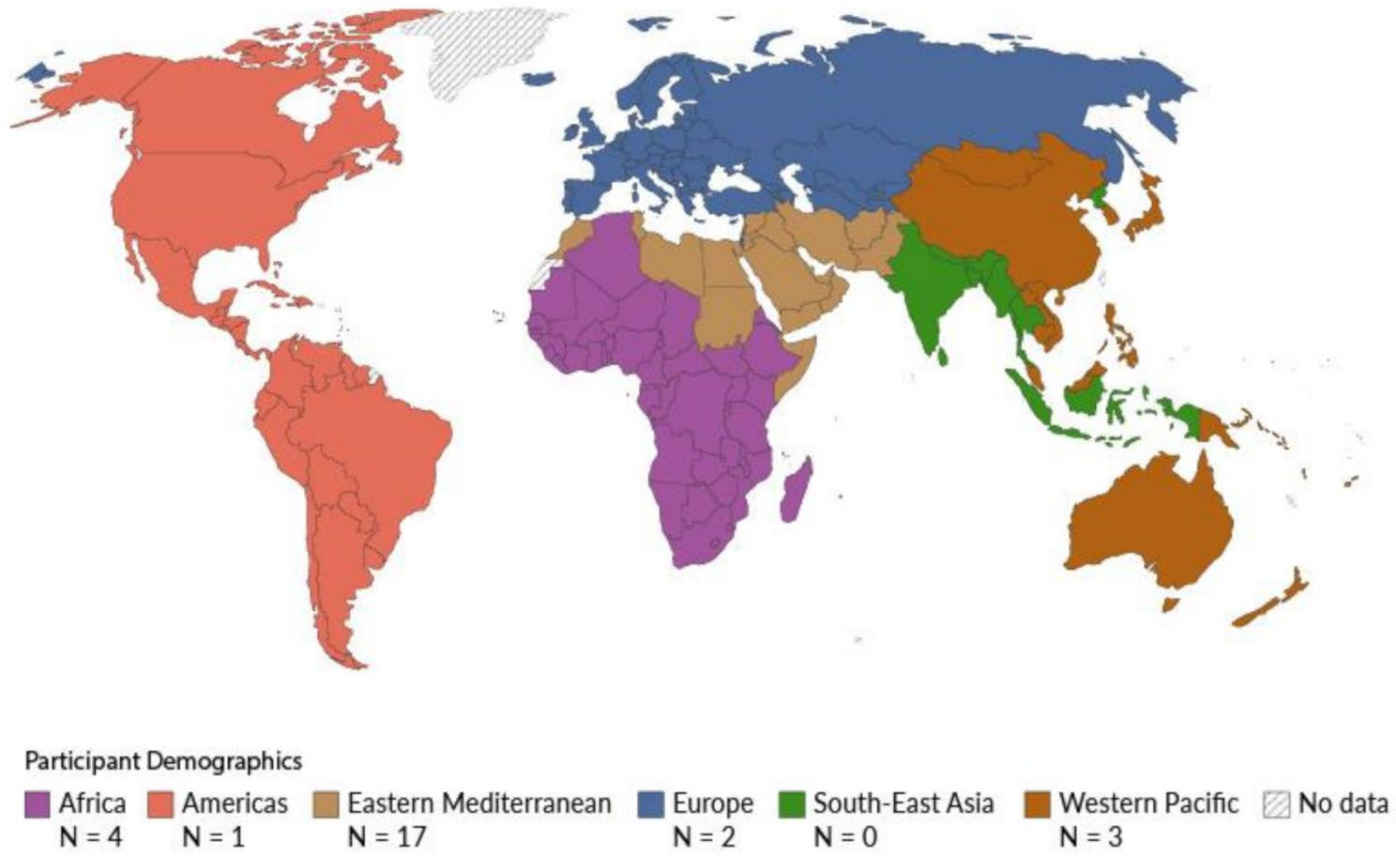
Participant countries of origin by WHO region *(Adapted from ‘Our World in Data’)* [26]

Participants’ demographics are presented in Table 1. The sample included 17 (63%) males and 10 (37%) females aged between 21 and 60 years. The majority were young (40.74% having between 30 and 39 years and 29.62% in the 18–29 years group). Participants originated from different WHO regions, and 17 different countries (Fig. 1) [26].

**Table 1:** Characteristics of participants (*N* = 27)

Variable	Characteristics	Frequency	Percentage (%)
Age	Range 21–60 (Median 33)		
	18–29	8	29.62
	30–39	11	40.74
	> 40	6	22.22
	Not Provided	2	7.40
Sex	Male	17	62.96
	Female	10	37.03
Ethnicity	Asian *	5	18.51
	Black **	5	18.51
	Mixed	1	3.70
	White	1	3.70
	Arab	10	37.0
	Other Not provided	4 1	14.8 3.70
Migration Status	Visa	7	25.92
	Asylum Seeker	5	18.51
	Refugee	3	11.11
	British Citizen	4	14.81
	EU Citizen	1	3.70
	Leave to remain	2	7.40
	Unclear/Prefer not to answer	5	18.51
Years in the UK	0–2 Years	14	51.85
	2–5 Years	6	22.22
	5–10 Years	2	7.40
	> 10 Years	5	18.51
Educational Status	No formal Qualifications Attained	3	11.11
	College/Technical	4	14.81
	Undergraduate/Bachelors	5	18.51
	Advanced degree***	10	33.33
	Other	5	18.51

*Asian category includes all those under Asian/Asian British united Kingdom census categories (Indian/Pakistan/Bangladeshi/Chinese/Other Asian)

** Black category includes all those under Black/Black British United Kingdom Census Categories (African, Caribbean, any other Black, African, or Caribbean backgrounds)

***Seven of these participants were pursuing advanced degrees at the time of the study

Comparing this demographic data with the latest Census 2021, we note that our study aligns with the overall characteristics of international migrants living in England and Wales– the majority are male, with similar ages and a significant proportion with ‘advanced degrees’ (33.3% in our study, compared to 44% in the Census Data) [27, 28].

### Qualitative data analysis– themes

Four main themes were identified during data analysis, as described below. These included: (1) Uncharted territory: the challenges of navigating a new healthcare system; (2) Preserving the sense of agency and decision-making around antibiotic use; (3) Self-perpetuating cycle and (4) The fragile state of the doctor-patient relationship.

#### Theme 1: uncharted territory: the challenges of navigating a new healthcare system

##### Access to care during journey

Some of the participants’ journeys to the UK, particularly the forcibly displaced migrants, involved travelling between different countries for extended periods of time, and many reported difficulties accessing healthcare and even basic facilities such as clean water along the way, which would have rendered them particularly vulnerable to contracting infections.

*“During my journey I had shortness of breath*, *but I wasn’t able to get any medical attention and I didn’t have medications” (P17*, *male*, *migrant status unclear)*.

*“It lasted from 10 to 15 days. I started from [place 1] (…) and went to another country. And I didn’t have any kind of healthcare and I didn’t have any kind of*, *even like clean water wasn’t available. I was scared during this journey” (P12*, *female*, *migrant status unclear)*.

##### Difficult access to healthcare in the UK

Participants spoke of how they struggled to book appointments telephonically with their General Practitioner (GP), with many reporting that the process was not user-friendly and relied on them to call the surgeries at specific times, otherwise they would be required to wait longer or try another day. If participants were successful in securing a consultation, many were surprised that their appointments were scheduled days in advance and were unable to see the doctor in the same day. In response to these delays, participants explained that they were self-medicating, using previously prescribed antibiotics to treat illnesses they would have visited the GP for.

*“I tried to access the GP. So I called them and the receptionist told us to call again and to call again and I couldn’t reach them*, *I couldn’t talk to a doctor. So I took the previous prescription and went to the pharmacy and got the same medication and I took this medication” (P17*, *male*, *migrant status unclear)*.

##### Comparison to healthcare systems between other countries and the UK

When asked how UK healthcare compared to their experiences accessing healthcare in other countries many participants felt it was quicker to access a doctor in other countries and that it was easier obtain a prescription, including antibiotics, as well as these being available over-the-counter:

*“The situation is very*, *very different from here. The doctors*, *of course they do prescribe the antibiotics whenever they feel it’s needed. But then it’s easily accessible in the pharmacy” (P2*, *female*, *visa-holder)*.

*“In our country it’s a little easier because we can ring (…) because our GP knows every patient (…) we don’t need to do appointment*, *we just call - ‘I’m [name]. I need this prescription because I am ill’*, *like that. And ‘Ok*, *we’ll give you the prescription’ and doctor just sign and that’s it” (P25*, *female*, *EU citizen)*.

We also identified that in some cases antibiotics were available over the counter in local international shops without prescriptions within ethnic communities in the UK. As one participant described,

*“Last time I worried about my teeth because (…) I lost my filling in the teeth and it started to painful*, *and I thought maybe it’s infection inside you know. But here the GP*, *dentist didn’t have appointment. It’s hard here*, *and I bought here [in the UK] antibiotics just in case because I go to [travelling abroad] and I don’t know what will happen. It was*, *before*, *two days before traveling (…) We go to (…) our shops (…) They have a lot of medicine– antibiotics as well” (P25*, *female*, *EU citizen)*.

#### Theme 2: preserving the sense of agency and decision-making around antibiotic use

Some participants demonstrated their perceived need to store antibiotics left-over from previous prescriptions, bring antibiotics back from other countries, and share them amongst friends and family. It is relevant to note that the majority of participants had been in the UK for less than two years, so it’s likely that many of the practices around antibiotic use would still be related to the type of experience they would have had in their countries of origin.

*“I have antibiotics from my country. I haven’t used it*, *they are there (…) because here (…) nobody gives you antibiotics” (P1*, *female*, *visa-holder)*.

*“Most people*, *they book for a doctor but sometimes you [share] the antibiotics*, *like if it’s the same problem*, *the same symptoms that you can give to a friend or family member as well (…) sometimes it will be difficult for the doctor. It [takes] a long time. So this is a shortcut (…) even sometimes you have some financial problems (P22*, *male*, *asylum seeker)*.

Others, however, felt that sharing of antibiotics may not be appropriate:

*“Unfortunately*, *I noticed that it’s a very common thing that people do that… they said they have medications*, *and they said*, *if they tell their friends that*, *if you feel unwell*, *just call me*, *I will give you medications and part of these medications will be antibiotics*, *but I tried to talk to them and convince them that it’s not the right thing to do*, *because you cannot take any antibiotics without any medical advice” (P14*, *male*, *asylum seeker)*.

*“In general*, *I wouldn’t do it without medical guidance or without a doctor prescribing it for me. But yes it happens. And I’ve seen people*, *one of my relatives or just friends. I’ve seen them exchanging antibiotics as if they were exchanging some*, *kind of*, *a drug. Yeah I’ve seen it but I’ve never done it personally” (P9*, *male*, *visa-holder)*.

Participants expressed feeling reassured when having antibiotics stored at home, which gave them a sense of support and being *“equipped for those difficult times*”. Most of them attributed their need to store antibiotics for possible future difficulties in accessing healthcare or seeing a doctor when they are feeling unwell. Participants who had antibiotics in their possession expressed these were only for “emergency use” and some mentioned that the packets were unopened or expired.

*“It can be delayed in accessing healthcare. So with my medical experience I decided to just get*, *I came alongside me with a pack of ciprofloxacin tablets and also metronidazole. I just kept it just in case I need it for anything”* (P6, male, *visa-holder)*.

Participants also described ways in which they achieved a sense of control in relation to their antibiotic use, such as having low thresholds for asking for antibiotics, and when antibiotics were prescribed, some participants talked about not finishing the full course of treatment when they felt well, preserving their sense of agency around when they needed antibiotics and how long they needed them for.

*“Whatever symptoms I have for some kind of cold or fever*, *when I take that medicine I mean for myself the first thing I do*, *I ask for antibiotics. I rely on medicine. There are people there who say to you*, *for example*, *‘No*, *I don’t need to take medicine for two or three days and after that I’ll be fine’*, *but for me I prefer to take the medicine; it makes me feel more better and relaxed” (P3*, *male*, *British citizen)*.

*“I stop taking the antibiotic when I feel better*, *and when my condition improved*, *I (won’t) keep them*, *I always trash them” (P14*, *male*, *asylum seeker)*.

#### Theme 3: Self-perpetuating cycle

A recurrent theme that was identified in the data was that many would have repeated courses of antibiotics due to certain factors (not necessarily related to clinical need), creating a ‘self-perpetuating cycle’ of antibiotic overuse. There were three main factors identified: (a) co-infections, (b) using alarming symptoms or “red-flags”, and (c) recurrent or persistent symptoms.

##### Co-infections

A few participants had knowledge around the risk of a superadded bacterial infection, when suffering from covid, for example, resulting in the need for antibiotics.

*“The doctors here*, *when you had covid. Do you need antibiotics for– they said no*, *but if it starts to*, *I think side effect from covid*, *the chest infection. So like when the lungs stopped work*, *so you*, *example*, *you need antibiotics. You know what you need. It’s not mean that you need to take the antibiotic because you have a covid– side effect from covid” (Migrant 25*, *female*, *EU citizen)*.

##### Using alarming symptoms or “red-flags”

Participants demonstrated that they used “red-flags” to get their GP to prescribe antibiotics for them. These “red-flags” included knowledge about specific symptoms relevant to bacterial infections, for example the colour of phlegm. Others expressed certain terms and *‘knowing what to say’* to get their GPs to prescribe them an antibiotic.

*“If you have like green muggles*, *if your nose*, *you need antibiotics. That is the thing. So if you say that symptom*, *you need antibiotics*, *which cause it’s not a viruses*, *it’s bacteria. I don’t know if that is true. I think that it’s no. But if you say that and you living in my country*, *you know what to say to have the antibiotics. So that’s one of the things that you need to say” (P1*, *female*, *visa-holder)*

*“I went to the GP and I told him it’s a cough and they usually say something*, *like ‘Oh no you don’t need an antibiotic’*, *And I’ll say ‘No*, *no but its producing phlegm etc’ or ‘What colour is the phlegm?’ And if you go through all the motions he’ll give you the antibiotics in the end” (P8*, *male*, *British citizen)*.

##### Taking antibiotics due to previous similar symptoms or persisting symptoms

Some participants justified expecting antibiotics or deciding to take antibiotics even without a prescription, when symptoms were similar to those experienced in the past or when symptoms persisted after a given period of time:

*“If I have the similar symptoms*, *I would take them [antibiotics] without any prescription*, *according to the same instruction that the doctor gave me on the previous one” (P20*, *male*, *refugee)*.

*“Because I used to (…) when I have these symptoms*, *I take antibiotics back home” (P1*, *female*, *visa-holder)*.

#### Theme 4: the fragile state of the doctor-patient relationship

Participants’ experiences of their interactions with healthcare professionals, in particular GPs, were quite varied. Some reported a general positive opinion, mentioning the use of interpreters or how practitioners adapted the pace of their consultations and the terms used to accommodate for difficulties in communication. This helped boosting trust between clinicians and patient:

*“They are really friendly. Also they make sure that you understand what’s going on and you understand your condition. They try to explain it to you in*, *like*, *normal terms and not medical ones just so the respondent or the recipient*, *myself in that case*, *understands what’s going on even if they do not have a medical background” (P9*, *male*, *visa-holder)*.

*“Yeah*, *there was a time I needed them [antibiotics] but then the doctor says to me ‘don’t take it’ or ‘I advise you to not take it*, *but if you take it it’s your own risk (…) so once he says it’s by your own risk*, *that just means you don’t take it and you listen. Follow your doctors*, *you know (…) I mean the doctor knows best what’s for me so I feel OK about it” (P3*, *male*, *British citizen)*.

However, in other cases there were clear barriers, either due to language difficulties, cultural differences or specific expectations in terms of care (for example, having a face-to-face examination, rather than a telephone call). Specifically in relation to antibiotic prescribing, some participants did not trust the doctor’s decision not to issue antibiotics and felt the need to acquire them elsewhere:

*“They just give me the same big no. So maybe if they’re like a little more. I don’t know more…[empathetic? ] will be better*, *and I didn’t have to reach another antibiotic for another way” (P1*, *female*, *visa-holder)*.

*“I didn’t finish the course because it was a phone consultation and I didn’t get examined*, *the doctor didn’t examine me and unfortunately his problem isn’t solved yet and they gave me another appointment” (P11*, *male*, *asylum seeker)*.

Where antibiotics were prescribed, participants interpreted this as their condition being particularly serious and felt scared when they had to take this type of medication:

*“I just feel I am in a very bad state*, *otherwise I wouldn’t have been prescribed antibiotics*, *but not really*, *and like not specifically feeling good about it. I just*, *I feel scared to be honest (…) just because the situation has come to a level of infection. That’s the main thing” (P2*, *female*, *visa-holder)*.

## Discussion

### Summary

This study has found that many migrant populations face multiple challenges in accessing healthcare, some whilst in transit from their country of origin, but the majority after arriving in the UK as well. In general, participants felt dissatisfied with the UK health service, citing examples such as complicated processes for arranging GP appointments, long waiting times and reluctance of doctors to prescribe antibiotics compared to what they had experienced before. Many migrants were used to purchasing antibiotics over-the-counter in their country of origin and for some, sharing antibiotics with friends and family or saving tablets for future use, was common practice. For many participants, being able to have antibiotics without needing a prescription or having them stored at home, reinforced their sense of autonomy and safety.

There were some factors identified as perpetuating the tendency for the overuse of antibiotics. Participants would often feel the need to exaggerate symptoms to get a prescription or would request antibiotics if they experienced similar symptoms as before or if their symptoms persisted after a certain period of time. In terms of the migrant-doctor relationship, several barriers were identified, including mistrust regarding the decision to prescribe antibiotics or not, cultural differences and language difficulties.

### Comparison with existing literature

Though there is a gap in research focused on factors contributing to antibiotic resistance among migrants, this work echoes previous studies on migrants’ experiences of antibiotics and access to healthcare in the UK. For example, Lindenmeyer and colleagues [30] found that newly arrived migrants were able to easily access antibiotics over the counter in their countries of origin and felt that in the UK there was often a delay in prescribing antibiotics, which could lead to medical complications. There were also references to a breakdown in communication between migrants and their GPs and they often felt their concerns were not taken seriously. Other studies reached similar conclusions regarding the overall mistrust and dissatisfaction with the UK healthcare system and GPs in particular [9, 31, 32].

These findings should raise significant concerns for the ongoing care of these populations, which is likely to affect other health-related issues that go beyond antibiotic prescribing. Anticipating previously encountered barriers, migrants may choose to seek help elsewhere [30], which can include bringing antibiotics back to the UK after visiting their countries of origin or trying to purchase them in the UK, as these seem to be available within certain international community shops as we found in this study. This may or may not help in the short-term, depending on whether antibiotics were indicated in the first place or not. In certain cases, delays in seeking help from a medical practitioner may make conditions deteriorate unnecessarily, as the right diagnosis is not reached and treatment not delivered in a timely manner. Additionally, for those individuals who choose to take antibiotics for previously encountered or persisting symptoms (which may in both instances be viral infections), this can lead to unnecessary, repeated courses of antibiotics, putting them at an increased risk of AMR.

Indeed, even for clinicians, diagnosing a viral or bacterial infection may be challenging, as symptoms can present very similarly, and there is often lack of objective diagnostic tests especially in primary care. In patients infected with covid-19, even previously useful tests (such as C-reactive protein) were found to be non-specific and unhelpful to differentiate them from a bacterial infection. There is also the risk of co-infections, which may at times be overestimated, as it seems to have been the case at the start of the pandemic, and lead to unnecessary antibiotic prescriptions [33]. Another factor could be the recent changes in delivery of care - particularly since the pandemic, as the number of remote consultations in primary care has increased, which may make clinicians want to avoid taking risks and issue antibiotics ‘just in case’ [34].

In terms of the healthcare system in general, it is not surprising, given the widely reported challenges facing the NHS, that migrants are experiencing many problems when trying to book an appointment with a GP or a dentist. This is clearly affecting different patient groups in the wider society, but the inherent limitations of the system become even more problematic when we add cultural differences in understanding how the system works and communication problems, to name just a few examples. Arguably, current processes do not take migrants’ specific experiences and needs into account and may be exacerbating existing disadvantages.

### Implications for research and practice

Trying to develop an in-depth understanding of the factors that could contribute to the risk of AMR among migrants is a good start to help address AMR and wider health disparities. As this paper has demonstrated, aligning with other research in this area, the factors are complex and multi-layered, involving the individual migrant, their wider communities, the particular relationship with healthcare professionals and broader systemic factors [35]. It would seem that future efforts to improve health outcomes in migrants should therefore focus on these different aspects. It will also be important to increase our knowledge on clinician’s experiences, what factors from the patient-doctor relationship influence antibiotic prescribing in certain migrant groups and whether this differs from that of the general population. The lack of trust experienced by some migrant groups, either being subtly or more overtly shared during consultations, as well as other difficulties such as time and communication problems, may affect the clinician’s decision around management and the threshold for starting antibiotics. Another important area of interest would be exploring the impact of the pandemic and changes in clinical systems, triage and remote consultations, given that certain migrant groups are known to have been disproportionally affected by covid-19.^36, 37^

Additionally, clinicians need to be made more aware of research findings that relate to migrant health, AMR, and their consultation skills and how best to improve this. Even within the limits of short consultations, there are aspects which could be addressed, such as clearer explanations of why antibiotics are being prescribed or not, the risks associated with over-prescribing and what alternative methods could help to alleviate symptoms.

Currently, AMR public health promotion and education campaigns implemented by the UK government’s UK Health Security Agency (UKHSA, formerly Public Health England) and the NHS lack specific tailored and culturally sensitive messaging to both minoritised ethnic and migrant communities in the UK. The study findings provide robust evidence in support of strengthening the need for both policy and implementation for holistic approaches to addressing AMR knowledge, attitudes and behaviour via health promotion and education campaigns in diverse migrant communities in the UK. Additionally, the results also demonstrate that improved access to primary and secondary care for migrants in the UK is vital for reducing AMR in migrant and ethnically diverse communities. Based upon the results and findings from this research study we have outlined a number of policy recommendations as shown in Table 2.

**Table 2:** Policy implications and recommendations

(1) *Health Promotion and Education* a. Consider the health needs of migrant populations and communities in the design and implementation of AMR health promotion interventions. Specifically, we recommend that UK government agencies, NHS, social care, and third-sector non-governmental organizations incorporate customised messaging to accommodate various language requirements of diverse communities. Additionally, these interventions should align with the cultural and religious needs of these various migrant communities, ensuring acceptability and effectiveness in the deployment of these campaigns. b. Include minoritised ethnic and migrant communities in the development of AMR health promotion interventions and awareness raising to ensure improved and sustainable uptake, as well as increased awareness of AMR and inappropriate use of antibiotics. c. Prioritise and increase funding for research to identify new approaches to strengthening engagement with migrant communities to improve health education and AMR awareness. d. Support research to evaluate AMR interventions and health promotion campaigns to assess their appropriateness and effectiveness for migrant communities, and inform evidence-based changes to policy and practice. (2) *Primary and secondary healthcare* a. Longer and tailored primary care appointment times to accommodate for translation and complex health needs, and to support knowledge transfer and patient education around AMR for ethnically diverse and migrant communities. b. Increase provision of and routinely evaluate translation and interpreting in health services to help counter language barriers and support patient health knowledge. We also advocate for increased training and awareness for healthcare staff of the availability and accessibility of these translator services during clinical appointments. c. Prioritise a holistic approach, addressing social determinants of health which contribute to the risk of AMR in migrant communities, including living conditions, deprivation, and access to care. d. Academic research partnerships and funding to support collaborative research with migrant communities to address the health improvement needs with regards to AMR.	

Finally, although the focus of this study was not to provide specific details of a particular ethnic or cultural group, clearly there will be differences in experiences based not only on the migrant’s background and country of origin but also the reasons behind their migration, the conditions of their journey and their overall adaptation and experience of the host country. There is an argument to keep findings on a more general level, to avoid further stigmatisation of already vulnerable groups. However, future interventions and antibiotic stewardship campaigns may be more successful if they are specifically targeted and take into account each group’s characteristics and circumstances. This may also require a better understanding of the attitudes and use of antibiotics in various world regions, as arguably this could influence migrants’ behaviour and expectations when they arrive in the UK.

More research work should also be aimed at understanding the specific dynamics of doctor-patient relationships in the case of migrant populations and what may be the drivers of inappropriate antibiotic prescription.

Research teams would also benefit from adopting an interdisciplinary approach, both in terms of their methods and hypotheses, which may help gain a deeper understanding of these groups and the intersectionality behind possible health inequalities.

Findings from the present work have helped shape other ongoing projects involving some of the co-authors– the ARK-EM project (**A**ntimicrobial **R**esistance and **K**nowledge through **E**ngagement with **M**igrant communities) aims to ascertain if, how and why antibiotic knowledge and antibiotic use may differ amongst different ethnic minority communities, and whether these differ from the ‘white majority group’. The methodology includes a systematic review^38^ and community engagement workshops.

### Strengths and limitations

The research team included people from various backgrounds, most of them ‘migrants’ themselves, which may have helped generate meaningful discussions with participants and contextualise the data during the analysis. Methodological issues that may have arisen were addressed through regular team discussions, including with the Project Advisory Committee.

Some of the migrants seemed at times reluctant to open up about their experiences, particularly about their journey to the UK and living conditions, as they felt that this information could negatively impact their immigration status. The research team tried to reassure participants explaining about confidentiality and anonymity and that they were free to share whatever they felt comfortable with.

Regarding the technical quality of the recordings, this was compromised in some of the interviews conducted remotely, which may have affected the clarity of short sections of the conversation. However, offering the possibility of online and phone interviews meant that many more participants were able to take part in the study and their opinions heard, which wouldn’t have been the case if only face-to-face interviews were offered.

## Conclusion

This research highlights existing challenges faced by migrants when trying to access healthcare services in the UK and difficulties emerging from consultations in primary care. These may partly explain practices around antibiotic use and should be taken into consideration in future policy and research initiatives (cf. Table 2: *Policy implications and recommendations*). More opportunities for dialogue should be created between clinicians, researchers, policy-makers, and migrant communities in order to plan realistic and effective interventions to prevent AMR in migrant populations.

## Electronic supplementary material

Below is the link to the electronic supplementary material.

Additional file 1: COREQ Checklist

## Data Availability

The datasets used and/or analysed during the current study are available from the corresponding author on reasonable request.
